# Using a Bayesian meta-frontier approach to evaluate airline performance: The nexus between airline efficiency and customer satisfaction

**DOI:** 10.1016/j.heliyon.2023.e20391

**Published:** 2023-09-22

**Authors:** Abdelrahman Rashid Bin Taliah, Panagiotis D. Zervopoulos

**Affiliations:** a University of Sharjah, College of Business Administration, Sharjah, United Arab Emirates; b University of Sharjah, Department of Management, Sharjah, United Arab Emirates

**Keywords:** Airlines, Efficiency, Customer satisfaction, Data envelopment analysis, Bayesian methods

## Abstract

A balanced panel of 83 airlines during 2011–2019, classified into (a) high perceived service quality and (b) low perceived service quality, was incorporated into the Bayesian meta-frontier framework. Our findings were obtained from applying a novel method to data sourced from the ICAO Data+ and Skytrax databases. We obtained consistent efficiency and meta-technology efficiency estimates, shedding light on airlines’ business models, products, and services to improve their efficiency and customer satisfaction. Specifically, we did not identify a trade-off between customer satisfaction and airline efficiency. However, larger firms were more likely to achieve this dual objective. Airlines classified as having high service quality reported greater allocative inefficiency than their low-quality service counterparts. During periods with or without minor external shocks (e.g., 2011–2019), airlines could offer premium products at reasonable prices or with a better quality and cost-of-service balance (e.g., premium economy cabins, extra legroom, inflight entertainment, and Internet).

## Highlights

• We estimate 83 airlines' efficiencies between 2011 and 2019.
• Airlines are classified into high and low perceived service quality.
• Bayesian data envelopment analysis is introduced in a meta-frontier framework.
• Trade-off between efficiency and customer satisfaction is not present for airlines.
• Airlines could emphasize premium products at reasonable prices.

## 1 Introduction

### 1.1 The impact of external shocks on airlines operations

The airline industry plays a significant role in the service sector. Services account for approximately two-thirds of the world's output and are projected to continue growing [1]. Although the airline industry has a significant impact on the global economy, aviation is vulnerable to various external shocks, such as financial crises, pandemics, climate change, and terrorism [[2], [3], [4], [5]]. For instance, the aviation industry was negatively impacted by the September 2001 terrorist attack on the World Trade Center Twin Towers, leading to a 20% drop in air travel [6]. In addition, terrorist-related threats in 2006 forced massive closedowns at British airports, disrupting the schedules of direct international and connecting flights [7]. Before the COVID-19 pandemic, viruses, such as severe acute respiratory syndrome (SARS), Middle East respiratory syndrome (MERS), and Swine Flu (H1N1), revealed the airline industry's vulnerability to global health crises, alongside the shock to the aviation sector of the Global Financial Crisis of 2008 [8]. Airlines recovered rapidly from the financial crisis with economic stimuli from governments [9]. According to Ryley et al. [10] and ICAO CAEP Aviation and Climate Change Factsheet (https://www.icao.int/environmental-protection/Documents/Factsheet%20Business%20and%20Economics%20Final.pdf), climate change is another threat to the airline industry. For instance, ash in the atmosphere from Iceland's volcanic eruption in 2010 interrupted flights between Europe and the US [11], while weather volatility due to climate change is expected to affect all aspects of flight operations (ICAO CAEP Aviation and Climate Change Factsheet).

According to Forsyth [8], exogenous shocks, such as those outlined above, lead to market adjustments in which some carriers modify their route maps and others fail and exit the market. Although low-cost carriers (LCCs) are expected to cope better with adjustments than full-service carriers (FSCs) because of their more efficient business models, it is not clear whether the former or latter will adapt better and be more sustainable [8].

### 1.2 Airline business models

Airlines are mainly classified as conventional or FSCs (sometimes called network carriers or legacy carriers) and LCCs [12]. The two strategies are very distinct [13]. However, FSCs commonly create their own LCC. For instance, LCC Transavia is a subsidiary of the FSC KLM, SAS operated Snowflake LCC (it ceased operations in 2004), Lufthansa formed the LCC Germanwings that now operates under the name Eurowings, and many other cases. This diversification strategy attempts to expand the FSCs’ market by entering new segments served by LCCs. According to Graf [14], a few dual-operation airlines have successfully operated LCC. Although LCCs started operating in the 40s in the US, since the 90s, the aviation industry has seen a considerable rise in LCCs [15], such as JetBlue (US), EasyJet (Europe), WestJet (Canada), Ryanair (Europe), and IndiGo (India). The low-cost strategy implemented by LCCs has transformed the market, leading to the adoption of FSCs, such as Aer Lingus [16].

Typically an FSC's objective is to provide “air transportation infrastructure,” connecting capitals and key economic and highly populated cities. In the last century, most FSCs were “Flag Carriers” [17], meaning they carried the country's flag and usually the name of a country or city. They are also responsible for developing a sophisticated global network outside their hub [18]. FSCs usually offer multiple seat configurations in their fleet, full meals, snacks, advanced entertainment systems onboard, and even telephone and internet services for their passengers. FSCs can extend their services to customers before and after flights, including chauffeur services for executive-class passengers, special check-ins, and lounge areas. Similar to Emirates and Turkish Airlines, FSCs have successfully used new airline technologies to create a hub connecting almost all seven continents [19].

Unlike FSCs, LCCs adopt a cost leadership strategy in which all frills are stripped out, and LCC customers are charged only for consumed services [[20], [21], [22]]. This strategy, which is known as the “strategy of commoditization” [23], reduces costs for airlines, allowing them to offer lower fares for the routes they operate [24]. For instance, Morrison [25] indicated that Southwest passengers in the US saved at least three billion dollars in 1998. LCCs focus on point-to-point travel, avoid connecting passengers, use a single-plane type with a single-type configuration, operate at secondary airports where possible, and emphasize direct sales without intermediaries [26]. The introduction of LCCs has been proven to enhance tourism traffic [17].

### 1.3 The nexus between airline efficiency and customer satisfaction

Duygun et al. [27] highlighted that the airline industry is asset-intensive and highly competitive. They also argued that both efficiency and customer satisfaction are key elements of success. This dual objective has not received sufficient attention in the literature on airlines. Considering customer satisfaction as important as efficiency, we classified airlines based on customer reviews of carrier service quality. Specifically, based on Skytrax World Airline Rankings, we construct two groups: (a) carriers consistently listed in the world's top 100 throughout the review period and (b) carriers not listed in the world's top 100 throughout the review period. We measure group-specific efficiency and meta-efficiency (i.e., the relative efficiency of all sample airlines across groups) for the period 2011–2019. The classification of airlines and use of the meta-frontier framework aims to attribute differences in the performance of the two airline groups based on their knowledge characteristics and strategies [28]. Skytrax World Airline Rankings are widely used measures of customer satisfaction in the airline industry [[29], [30], [31], [32]]. The classification of airlines based on customer satisfaction is crucial for answering the two research questions of this study, which are articulated in Subsection 1.4.

### 1.4 Novelty and research questions

We acknowledge that group-specific efficiencies and meta-efficiencies are biased because of the limited sample size and dimension of the input-output space. To address this issue and obtain consistent estimates, we used a novel Bayesian generalized directional distance function data envelopment analysis (DEA) approach [33]. This study aims to identify business models that can simultaneously improve airlines' efficiency and customers' perceptions of the quality of airlines’ products and services. Our findings provide practical suggestions for decision-makers.

The relationships between customer satisfaction, airline operational performance, and profitability have been studied [[34], [35], [36], [37], [38]]. The extant literature includes various, and sometimes contradictory, findings regarding these relationships. For instance, Anderson et al. [39], Kamakura et al. [40], Banker et al. [41], and Zervopoulos et al. [42] argue that an inverse relationship exists between customer satisfaction and firm efficiency, while efficiency is directly related to profits. Suzuki [34] identified a tradeoff between service quality and airline profits. Meanwhile, Davis-Sramek et al. [36], Heskett et al. [43], and Loveman [44] justified a positive effect of service quality and customer satisfaction on firm profitability. A third stream of literature [37,38] reveals the moderating role of airline strategy (i.e., LCCs and FSCs) in the relationship between service quality and airline profitability. In other words, the direction of this relationship depends on an airline's strategy. Specifically, the impact of service failure (the inverse of the quality of service) is negative for LCCs, but it may not affect FSCs' efficiency and profitability.

This study adopts a different approach compared to others found in the literature (*see the paragraph above*), in which airlines are classified not according to their strategy (i.e., LCC or FSCs) but rather customer satisfaction level (i.e., (a) high perceived service quality and (b) low perceived service quality carriers). In addition, customer satisfaction is directly linked to airlines’ technical and allocative (in)efficiency through a meta-frontier analysis, in which the quality and cost of airline services are evaluated simultaneously. To the best of our knowledge, this is the first study to use consistent airline efficiency estimates obtained from a novel Bayesian approach to shed light on the links among airline service quality, technical efficiency, and the cost of service (allocative efficiency). The group-specific and meta-efficiency estimates used in this study were bias-corrected, minimizing distortions due to the sample size and dimension of the input-output space. Consequently, the findings and managerial implications of this study are considered valid.

The research questions (RQ) addressed in this study are as follows.

RQ1 What is the relationship between perceived quality of services and airline efficiency?

RQ2 How does the allocative efficiency of airlines shape their strategy toward customer satisfaction and profit growth?

### 1.5 Roadmap

The remainder of this paper is organized as follows. Section 2 reviews the literature on efficiency measurement and customer satisfaction in the aviation sector. Section 3 presents the methodology, which is a novel Bayesian DEA approach incorporated into the meta-frontier framework. Section 4 presents the selected variables, which are justified by relevant literature and the descriptive statistics of the dataset. Section 5 discusses the results of the empirical analysis and provides managerial implications. Subsection 5.1 links the findings of this study with the extant literature, some of which have not been discussed in previous papers, and provides managerial implications. Section 6 concludes.

## 2 Literature review

Airline efficiency has been studied intensively since the 60s [45]. History has shown that high fuel price volatility, global crises, and climate change have given airlines no option but to closely and strictly monitor their efficiency [46]. Governments have tried to intervene and regulate airlines' fares (e.g., ticket price floors) to protect their flag carriers; however, this has often negatively impacted airlines' efficiency. According to Douglas and Miller [47], airlines can optimize their efficiency in free markets. By emphasizing airlines’ efficiency, LCCs have revolutionized the aviation industry and set new efficiency standards [45,48]. Specifically, airline market deregulation, including removing protectionism on flag carriers and lowering entry barriers, has increased competition in the airline industry, improved airline efficiency, and allowed the entry of LCCs [49]. Airline market deregulation has strengthened other industries such as tourism [50] and improved passenger satisfaction [51].

Airline efficiency improvement is not a straightforward process and is primarily determined by endogenous factors. Forsyth et al. [52] point out that FSCs are government monopolies that have become publicly listed companies. Low and Lee [53] extended the discussion on airlines, emphasizing that they operate in highly competitive markets and experience competition from their international counterparts, while regional and global regulations are in place. Forsyth et al.‘s [52] outline of the aviation industry, in conjunction with Douglas and Miller's [47] discussion of airlines' efficiency optimization, justifies the challenges in improving airlines' performance. Saranga and Nagpal [54] highlighted the critical role of performance measurements in airline success.

DEA is the most widely used method to measure airline efficiency [55,56]. Various DEA expressions have been used in the extant literature, such as conventional constant and variable returns to scale DEA [32,46,[57], [58], [59]], super-efficiency DEA [56], and Malmquist DEA [60]. All the aforementioned studies regard airlines' operations as a “black box,” where inputs are introduced in a unified process to produce outputs. A growing body of airlines' efficiency literature utilizes network data envelopment analysis (NDEA) models that consider the internal structure of firms' operations. Specifically, NDEA models facilitate the measurement of a firm's overall efficiency, as well as the efficiency of its sub-processes or stages [[61], [62], [63]]. In the literature on airlines, most network structures unfold into two stages [[64], [65], [66], [67]], although a three-stage structure is also used [[68], [69], [70], [71], [72]]. The third stage represents sales efficiency, whereas the first two stages refer to operations and services.

Another strand of literature emphasizes two-stage approaches, combining DEA with regression models and data uncertainty. Two-stage models draw either on conventional or stochastic DEA approaches to obtain efficiencies (e.g., bootstrap DEA) and Tobit regression or generalized least squares to regress environmental factors on efficiency [46,[73], [74], [75]].

Data uncertainty (e.g., data errors) as well as the dimensionality of the input-output space and sampling variations are responsible for efficiency distortions [76]. Barros and Peypoch [77] and Omrani et al. [78] acknowledge the possible bias of DEA efficiencies and use bootstrap DEA and a multi-objective best-worst robust DEA method, respectively, to improve the validity of efficiency estimates. Apart from the sensitivity of efficiency estimates, which this study addresses using a Bayesian DEA approach [79], have uncovered assumptions and points out some of the most significant DEA limitations associated with those assumptions. For instance, DEA assumes the homogeneity of the units under review, the use of common technologies, and the isotonicity of inputs and outputs (i.e., an increase in inputs deteriorates efficiency, while an increase in outputs improves efficiency). In addition, the selected variables should capture all activity levels.

An airline's emphasis on efficiency improvement and customer satisfaction is desirable [80]. The importance of customer satisfaction for a firm's long-term success has been well established in the literature [39,81]. Scheraga [82] and Ringle et al. [83] justified the central role of customer satisfaction in achieving airlines' corporate goals. Skytrax airlines' reviews are probably the most widely used ratings for measuring passengers' perceptions of the quality of the onboard product (e.g., seat comfort, cabin cleanliness, cabin lighting/ambiance) and cabin services (e.g., boarding assistance, service friendliness/hospitality, problem-solving skills) (https://www.worldairlineawards.com/awards-methodology/). Although achieving both customer satisfaction and efficiency is desirable, the potential tradeoff between these two objectives may be a severe obstacle for many firms, especially in service industries, such as airlines [39]. According to the same study, this inverse relationship between customer satisfaction and efficiency is more likely to exist when the perceived quality of services is customized rather than standardized. Considering the two main Skytrax rating criteria sets (i.e., onboard product and cabin services), airline service quality is regarded as a mix of standardized and customized services. Ringle et al. [83] demonstrate the role of perceived safety in commercial airline passenger satisfaction. An earlier study by Rhoades and Waguespack [84] argued that airline passenger satisfaction and perceived safety are directly related. According to Swaminathan et al. [80], customer satisfaction and efficiency are more likely to be attained by larger firms that generate more revenue than by smaller firms.

## 3 Methodology

The methodology used in this study draws on the Bayesian DEA approach developed by Zervopoulos et al. [33], which was introduced in a meta-frontier framework originally formulated by Battese and Rao [85], Battese et al. [86], and O'Donnell et al. [87].

Specifically, we use a Bayesian generalized directional distance function data envelopment analysis (GDDF DEA) approach to estimate efficiency. Acknowledging that efficiencies are upward biased in the presence of finite samples, which are regarded as inadequate for the dimensionality of the production set [76,88,89], we applied a Bayesian DEA bias correction method. It has been proven that this method yields consistent estimates with lower mean square error (MSE) than that reported by widely used approaches in the DEA literature [33], such as the smoothed bootstrap [90], a “hybrid” Bayesian DEA method [91], and the stochastic non-parametric envelopment of data (StoNED) [92,93]. The performance of the Bayesian DEA estimates for scaled real-world and simulated samples (i.e., sample sizes of 30, 50, 80, 100, 200, 300, 500, 1000, and 1500 units), as well as a comparative analysis of the alternative bias-correction methods for efficiency is available in Zervopoulos et al. [33].

Given that the airlines under review are classified into two groups (i.e., (a) airlines consistently listed in the world's top 100 based on Skytrax assessment and (b) airlines not listed in the world's top 100 throughout the review period), we obtain group-specific efficiencies by applying the following model:

$$\theta^{\gamma }=\min\frac{1-\frac{1}{m}\sum_{i=1}^{m}\beta^{\gamma }g_{i}/x_{io}^{\gamma }}{1+\frac{1}{s}\left(\sum_{r=1}^{s}\beta^{\gamma }g_{r}/y_{ro}^{\gamma }\right)}$$

$$s.t.\;\sum_{j=1}^{n}\lambda_{j}^{\gamma }x_{ij}^{\gamma }+\beta^{\gamma }g_{x}\leq x_{io}^{\gamma }\;i=1,\ldots ,m$$

$$\sum_{j=1}^{n}\lambda_{j}^{\gamma }y_{rj}^{\gamma }-\beta^{\gamma }g_{y}\geq y_{ro}^{\gamma }\;r=1,\ldots ,s$$

(1) $$\sum_{j=1}^{n}\lambda_{j}^{\gamma }=1$$

$$\lambda_{j}^{\gamma }\geq 0\;\gamma =1,\ldots ,\Gamma$$

$$g_{x}=g_{y}=1$$

where $\theta_{j}^{\gamma }$ refers to group-specific efficiencies ${\left\{\theta_{j}^{\gamma }\right\}}_{j=1}^{n}\in (0,1]$. Also, $x_{i}=\left(x_{1},\ldots ,x_{m}\right)∊R_{+}^{m}$ and $y_{r}=\left(y_{1},\ldots ,y_{s}\right)∊R_{+}^{s}$ express inputs and outputs, respectively, and $g_{x}$ and $g_{y}$ are the corresponding direction vectors. $\lambda_{j}^{\gamma }$ represent group-specific optimal weights assigned to inputs and outputs. The group-specific proportional decrease in inputs and increase in outputs are denoted by $\beta^{\gamma }g_{i}/x_{io}^{\gamma }$ and $\beta^{\gamma }g_{r}/y_{ro}^{\gamma }$, respectively.

The meta-technology is defined by the union of the group-specific technologies

(2) $$T^{meta}\left(x\right)=\left\{T^{1}\left(x\right)\cup \ldots \cup T^{\Gamma }\left(x\right)\right\}$$

where

(3) $$T^{\gamma }\left(x\right)=\left\{\left(y\right):x\;can\;produce\;\left(y\right)\right\}$$

Given expressions (2) and (3), the meta-efficiencies are measured as follows:

$$\theta^{meta}=\min\frac{1-\frac{1}{m}\sum_{i=1}^{m}\beta^{meta}g_{i}/x_{io}^{\gamma }}{1+\frac{1}{s}\left(\sum_{r=1}^{s}\beta^{meta}g_{r}/y_{ro}^{\gamma }\right)}$$

$$s.t.\;\sum_{\gamma =1}^{\Gamma }\sum_{j=1}^{n}v_{j}^{\gamma }x_{ij}^{\gamma }+\beta^{meta}g_{x}\leq x_{io}^{\gamma }\;i=1,\ldots ,m$$

$$\sum_{\gamma =1}^{\Gamma }\sum_{j=1}^{n}v_{j}^{\gamma }y_{rj}^{\gamma }-\beta^{meta}g_{y}\geq y_{ro}^{\gamma }\;r=1,\ldots ,s$$

(4) $$\sum_{\gamma =1}^{\Gamma }\sum_{j=1}^{n}v_{j}^{\gamma }=1$$

$$v_{j}^{\gamma }\geq 0\;\gamma =1,\ldots ,\Gamma$$

$$g_{x}=g_{y}=1$$

Models (1) and (4), which draw on Cheng and Zervopoulos [94], can easily be extended to accommodate undesirable outputs and negative values in the data. In addition, the efficiencies obtained from these two models were independent of the direction vector length and were consistent with conventional DEA efficiencies.

We employ a new Bayesian DEA approach to correct the bias of both group-specific and meta-efficiencies [28]. Specifically, let $\Theta ={\left\{\theta_{j}^{\gamma }\right\}}_{j=1}^{\kappa }\in [\theta_{L}^{\gamma },1)$ where κ⊂n and $\theta_{L}^{\gamma }\in \left(0,1\right)$ be uniformly distributed. Then, the likelihood function is maximized at ${\hat{\theta }}_{L}^{\gamma }=\min\;\Theta \;\left(0<\theta_{L}^{\gamma }\leq \theta_{j}^{\gamma }<1\right)$ where j=1,…,κ. The expected value and unbiased estimator of the parameter $\theta_{L}^{\gamma }$ are:

(5) $$E_{\kappa }\left\{{\hat{\theta }}_{L}^{\gamma }\right\}=\theta_{L}^{\gamma }+\frac{1-\theta_{L}^{\gamma }}{\kappa +1}$$

(6) $${\tilde{\theta }}_{L}^{\gamma }=\frac{{\hat{\theta }}_{L}^{\gamma }\left(\kappa +1\right)-1}{\kappa }$$

where $E_{\kappa }\left\{{\tilde{\theta }}_{L}^{\gamma }\right\}=\theta_{L}^{\gamma }$.

Assuming that $\theta_{L}^{\gamma }$ is beta distributed, the Bayesian prior reads as follows:

(7) $$f_{\theta_{L}^{\gamma }}\left(\theta_{L}|\alpha ,\delta \right)=\frac{1}{B\left(\alpha ,\delta \right)}\theta_{L}^{\gamma \left(\alpha -1\right)}{\left(1-\theta_{L}^{\gamma }\right)}^{\delta -1},\theta_{L}^{\gamma }\in \left(0,1\right)$$

where α and δ are the two positive shape beta distribution (B) parameters. It is well known that B is defined as

(8) $$B\left(\alpha ,\delta \right)=\int_{0}^{1}\eta^{\alpha -1}{\left(1-\eta \right)}^{\delta -1}d\eta$$

with expected value: $E\left\{\eta \right\}=\frac{\alpha }{\alpha +\delta }$ (9)

Assuming that the unbiased estimator of $\theta_{L}^{\gamma }$ equals to the expected value of the Bayesian prior, we obtain:

(10) $$E\left\{\theta_{L}^{\gamma }\right\}=\frac{\alpha }{\alpha +\delta }$$

Then, the parameter δ is defined:

(11) $$\delta =\frac{\left(1-{\tilde{\theta }}_{L}^{\gamma }\right)\alpha }{{\tilde{\theta }}_{L}^{\gamma }}$$

The joint probability density function of $\Theta ={\left\{\theta_{j}^{\gamma }\right\}}_{j=1}^{\kappa }$ is:

(12) $$f_{\Theta }\left(\Theta \right)=\int_{0}^{1}f\left(\Theta |\theta_{L}^{\gamma }\right)f_{\theta_{L}^{\gamma }}\left(\theta_{L}^{\gamma }|\alpha ,\delta \right)d\theta_{L}^{\gamma }=\frac{B\left(a,\delta -\kappa \right)}{B\left(\alpha ,\delta \right)}$$

where δ>κ. Consequently, a is bounded as follows:

(13) $$\alpha >\kappa \frac{{\tilde{\theta }}_{L}^{\gamma }}{1-{\tilde{\theta }}_{L}^{\gamma }}$$

The posterior beta distribution is written as follows:

(14) $$f_{\theta_{L}^{\gamma }|\Theta }\left(\Theta \right)=\frac{f_{\Theta |\theta_{L}^{\gamma }}\left(\Theta |\theta_{L}^{\gamma }\right)f_{\theta_{L}^{\gamma }}\left(\theta_{L}^{\gamma }|\alpha ,\delta \right)}{f_{\Theta }\left(\Theta \right)}=\frac{1}{B\left(\alpha ,\delta -\kappa \right)}\theta_{L}^{\gamma \left(\alpha -1\right)}{\left(1-\theta_{L}^{\gamma }\right)}^{\left(\delta -\kappa \right)-1}$$

The posterior is shifted to the right compared with the prior. In other words, the former was overestimated, whereas the latter was bias-corrected.

(15) $$E_{\kappa }\left\{\theta_{L}^{\gamma }|\Theta \right\}>E_{\kappa }\left\{\theta_{L}^{\gamma }\right\}\;\text{as}\;\frac{\alpha }{\alpha +\delta -\alpha }>\frac{\alpha }{\alpha +\delta }$$

The bias-correction parameter is as follows:

(16) $$\xi =\frac{{\tilde{\theta }}_{L}^{\gamma }}{{\hat{\theta }}_{L}^{\gamma }}<1$$

Using (11), (13), (16), we obtain

(17) $$\hat{\alpha }=\kappa {\tilde{\theta }}_{L}^{\gamma }/\left(1-\xi \right)$$

and

(18) $$\hat{\delta }=\left(1-{\tilde{\theta }}_{L}^{\gamma }\right)\hat{\alpha }/{\tilde{\theta }}_{L}^{\gamma }$$

We estimate α and δ using the MATLAB function *betarnd*.

The ratio (16) is best fitted by a normal distribution with parameters $\hat{\mu }$ and $\hat{\sigma }$, expressing the mean and standard deviation, respectively, using the MATLAB function *normfit*. The bias-corrected estimates ($\theta_{j}^{\gamma \left(c\right)}$) are obtained as follows:

(19) $$\theta_{j}^{\gamma \left(c\right)}=\omega^{-1}\sum_{\psi =1}^{\omega }{\overset{ˇ}{\theta }}_{j\psi }^{\gamma }$$

where ω denotes the number of Monte Carlo iterations (ω=1,000), and ${\overset{ˇ}{\theta }}_{j\psi }^{\gamma }$ are randomly generated from the MATLAB function *normrnd*, with parameters $\theta_{j}^{\gamma }\hat{\mu }$ and $\theta_{j}^{\gamma }\hat{\sigma }$. The efficiencies $\theta_{j}^{\gamma }$ are identified by program (1).

The bias-correction procedure described above refers to group-specific efficiency. The bias-correction procedure for the meta-efficiency was straightforward. By introducing meta-efficiencies into expressions (5)–(19), we obtain the corresponding estimates:

(20) $$\theta_{j}^{meta\left(c\right)}=\omega^{-1}\sum_{\psi =1}^{\omega }{\overset{ˇ}{\theta }}_{j\psi }^{meta}$$

The objective of the meta-frontier framework is to identify technology heterogeneity among the sample units (e.g., airlines) captured by technology gaps. By extending the work of Kounetas and Zervopoulos [95], we estimated the technology gaps as follows:

(21) $${TG}_{j}^{\left(c\right)}=\theta_{j}^{\gamma \left(c\right)}\times \left(1-{MTR}_{j}^{\left(c\right)}\right)$$

where MTR stands for meta-technology ratio [88], and it is estimated as follows:

(22) $$0<{MTR}_{j}^{\left(c\right)}=\frac{\theta_{j}^{meta\left(c\right)}}{\theta_{j}^{\gamma \left(c\right)}}\leq 1$$

According to the technology heterogeneity concept [87,95], technology gaps are caused by exogenous factors, such as (a) national economic conditions, (b) national regulations and legal framework, (c) structure of national markets, (d) national cultural profiles and social dynamics.

## 4 Sample and data description

This study draws on both operational factors and customers' perceptions of airlines' quality of services to identify the best-performing firms, the presence of a resilient business model, and whether passenger satisfaction affects airlines’ efficiency. The selected variables express four aspects of airline operations: resources, capacity, productivity, and profitability. The variables employed are similar to those used by Kuljanin et al. [96]. The number of airlines under review was 83, which were classified into two groups based on the perceived quality of services provided: (a) 47 high-perceived quality airlines and (b) 36 low-perceived quality airlines. Emphasizing the airline type in our analysis, 71 were FSCs, and 12 were LCCs. We acknowledge that FSCs commonly operate cargo businesses in addition to their passenger businesses, unlike LCCs. However, the source of our data (ICAO Data+) does not separate passengers from the cargo business for airlines. In addition, having both FSCs and LCCs in the same sample and comparing these two types of airlines is frequently reported in the literature [75,97]. Data availability for the variables throughout the review period (i.e., 2001–2019) was the main selection criterion for airlines. To prevent distortions in the outputs of our analysis, it is important that the selected period is not affected by significant external shocks, such as global financial crises or pandemics.

The operational variables used in this study unfold into two inputs (i.e., number of employees and available seat-kilometers (ASK)) and two outputs (i.e., operating revenue and revenue passenger-kilometers (RPK)). The number of staff members is a widely used input variable (resource) to measure airline efficiency [46,96,98,99], and [100]. ASK captures an airline's total flight passenger capacity and is a major driver of revenue generation. In the literature, ASK is commonly used as an input [58,96,98,99]. However, few studies have used the ASK as the output [66,68,101]. Studies in which ASK serves as an output employ network DEA models, in which the output is a linking activity between the two stages. Hence, the ASK produced by one stage is the input for the subsequent stage.

Two outputs (operating revenues and RPK) were regarded as proxies for profitability and productivity, respectively [96]. Specifically, RPK represents the air traffic volume [56]. Given that these two metrics express different aspects of airline operations, they are commonly used together as outputs [96,99,[102], [103], [104]]. The ICAO Data + database was used as the source of all operational variables.

In addition to the operational variables, we employed user satisfaction data to divide airlines into two groups: high- and low-perceived quality airlines. The source of customer satisfaction data is Skytrax. In this sense, airlines consistently ranked in the world's top 100 list are regarded as having high perceived quality, while those that are not placed on this list throughout the review period (i.e., 2011–2019) are deemed low-perceived quality airlines. It should be noted that the Skytrax World Airline Awards report was not released in 2020 owing to the COVID-19 pandemic.

The descriptive statistics of the variables for the airlines under review are available in Table 1 and the sample airlines are presented in Table A1 of the Appendix.

**Table 1:** Descriptive statistics.

Variables	Measures	2011	2012	2013	2014	2015	2016	2017	2018	2019	CAGR
Inputs		Group 1: High perceived quality services airlines									
Number of Employees	Mean	17,671.8	18,772.2	19,624.8	21,322.7	22,405.3	23,584.2	24,239.4	25,018.7	25,789.4	0.04
	St. Deviation	18,123.3	18,996.7	19,795.2	22,916.8	23,862.6	25,985.2	26,168.1	26,845.5	27,791.4	0.05
	Min	1,590.0	1,347.0	1,459.0	1,678.0	1,679.0	1,588.0	2,041.0	2,000.0	1,984.0	0.02
	Max	78,000.0	74,000.0	80,175.0	83,132.0	87,202.0	95,322.0	105,746.0	103,363.0	107,391.0	0.04
ASK (million)	Mean	55,595.1	59,903.6	63,482.8	67,310.2	71,808.4	76,647.9	80,189.0	84,176.8	87,058.3	0.05
	St. Deviation	49,707.9	54,323.1	57,768.1	60,610.1	65,845.1	71,409.2	74,039.2	77,359.5	78,932.4	0.05
	Min	1,878.0	1,993.0	2,103.0	2,299.0	2,409.0	3,286.0	4,580.0	5,145.0	4,687.0	0.11
	Max	194,137.0	226,625.0	264,365.0	288,393.0	324,464.0	361,433.0	375,823.0	389,467.0	345,403.0	0.07
Outputs											
Operating Revenues (million $)	Mean	6,884.8	8,256.1	9,117.1	10,676.0	10,120.8	9,597.0	12,265.4	10,157.9	10,727.3	0.05
	St. Deviation	7,105.2	8,311.7	9,126.0	13,696.5	10,917.5	10,446.0	15,621.7	11,770.8	12,435.7	0.06
	Min	547.6	582.5	563.1	685.8	610.5	105.7	596.2	635.6	595.6	0.01
	Max	31,893.0	35,230.0	38,287.0	75,910.0	40,815.0	40,421.0	66,970.0	44,538.0	48,063.0	0.05
RPK (million)	Mean	43,674.4	47,897.9	51,255.8	53,751.0	57,547.2	63,978.6	64,739.1	67,914.8	70,200.6	0.05
	St. Deviation	39,743.6	44,011.8	46,872.0	49,202.3	52,762.4	66,322.0	58,670.3	61,315.7	62,067.2	0.05
	Min	1,471.0	1,543.0	1,696.0	1,866.0	1,996.0	2,874.0	4,106.0	4,365.0	4,060.0	0.12
	Max	153,264.0	180,879.0	209,227.0	230,885.0	251,190.0	375,823.0	288,885.0	302,297.0	270,709.0	0.07
Sample size		47	47	47	47	47	47	47	47	47	
Variables	Measures	2011	2012	2013	2014	2015	2016	2017	2018	2019	CAGR
Inputs		Group 2: Low perceived quality services airlines									
Number of Employees	Mean	4,853.7	4,996.7	5,152.2	5,375.2	5,630.9	5,675.1	6,201.8	6,539.2	6,760.9	0.04
	St. Deviation	7,362.9	7,428.7	7,538.3	7,219.3	7,708.2	7,545.6	7,862.3	8,104.8	8,268.8	0.01
	Min	136.0	173.0	180.0	195.0	213.0	349.0	351.0	383.0	580.0	0.17
	Max	40,003.0	40,287.0	40,788.0	41,105.0	41,840.0	42,859.0	43,848.0	44,598.0	45,294.0	0.01
ASK (million)	Mean	10,467.9	10,810.5	11,333.9	12,270.2	12,920.1	14,271.3	15,788.7	16,247.1	18,486.3	0.07
	St. Deviation	21,554.5	22,218.9	22,972.4	23,977.9	24,653.5	29,043.8	32,011.4	32,209.1	36,747.0	0.06
	Min	490.6	459.3	350.3	328.3	317.3	601.6	609.2	789.3	814.0	0.06
	Max	114,025.0	118,388.0	124,472.0	125,296.0	124,472.0	151,534.0	164,525.0	178,288.0	191,451.0	0.06
Outputs											
Operating Revenues (million $)	Mean	1,899.7	2,092.8	2,040.5	2,805.2	2,396.7	2,615.4	2,635.9	3,065.0	3,170.4	0.06
	St. Deviation	2,850.1	3,169.9	3,240.0	5,158.7	4,347.8	5,034.7	5,403.4	6,856.0	7,402.2	0.11
	Min	47.9	50.3	48.4	49.3	51.6	40.2	42.8	50.2	56.1	0.02
	Max	11,372.0	12,859.0	15,580.0	23,750.0	22,491.0	26,055.0	30,948.0	40,266.0	43,522.0	0.16
RPK (million)	Mean	8,434.9	8,556.6	9,039.9	9,920.9	10,432.5	11,915.4	13,353.7	14,135.5	16,149.2	0.07
	St. Deviation	17,692.5	18,290.7	19,060.7	20,489.0	20,611.3	26,400.1	29,263.6	30,025.8	33,886.5	0.07
	Min	324.2	313.4	263.1	227.5	211.9	421.8	462.0	499.9	504.1	0.05
	Max	93,875.0	96,990.0	102,921.0	108,172.0	102,921.0	142,740.0	157,113.0	170,900.0	183,554.0	0.08
Sample size		36	36	36	36	36	36	36	36	36	

CAGR: compound annual growth rate.

All variables in Table 1 present positive compound annual growth rates (CAGR) for 2011–2019. The selected period spanned after the Global Financial Crisis and before the COVID-19 pandemic, justifying the increase in all selected variables. The growth rate for most of the variables associated with Group 2 airlines (i.e., low-perceived quality service airlines) was higher than that of Group 1. However, on average, the airlines classified into Group 1 are considerably larger than their counterpart Group 2 firms, as indicated by the variables in Table 1.

## 5 Empirical results

**Fig. 1:**
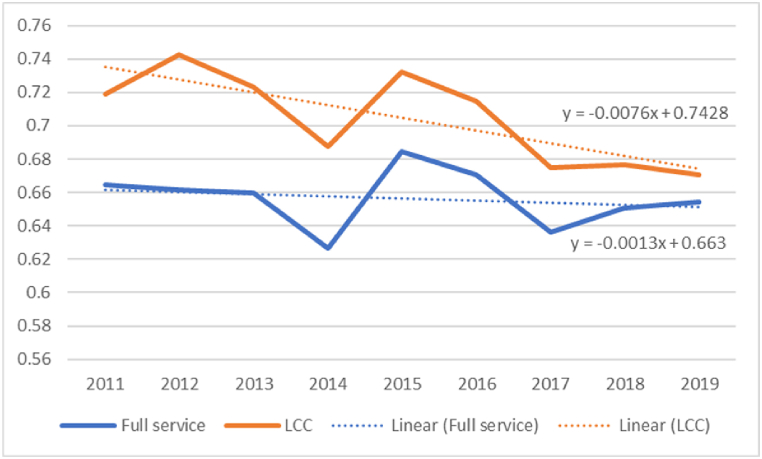
Efficiency estimates for FSCs and LCCs.

By incorporating all sample airlines (i.e., 83 airlines) into expressions (1) and (5)–(20),^1^ we obtain the bias-corrected efficiencies for FSCs and LCCs. At this point, we do not distinguish the sample airlines into high- and low-perceived quality services, but based only on their type, into FSC and LCC. Hence, we do not use the meta-frontier framework outlined in expressions (4), (21), and (22). At this stage, the reason for assessing all sample airlines' performances without considering the high/low perceived service quality classification is to test whether our outcomes are in line with the theory that claims that LCCs are more efficient than FSCs [8]. Indeed, on average, the sample LCCs were assigned higher efficiencies than FSCs throughout the review period (Fig. 1). We should highlight that the selected period (i.e., 2011–2019) is free from external shocks such as the Global Financial Crisis and COVID-19. Irrespective of the better performance of LCCs compared to FSCs, the efficiency of both types of airlines dropped over the period 2011–2019. In particular, LCCs’ efficiency reports a negative compound annual growth rate of 0.76%, whereas the efficiency of FSCs declines, on average, by 0.18% (Table A2 in the Appendix).

**Fig. 2:**
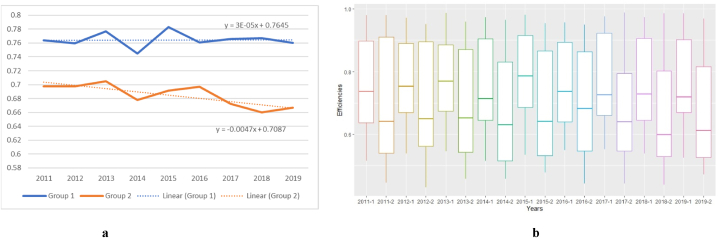
aGroup-specific efficiencies, Fig. 2b. Group-specific efficiencies boxplots Fig. 2. Group-specific efficiencies for high (Group 1) and low (Group 2) perceived quality services airlines.

When the sample airlines were classified into high perceived quality (Group 1) and low perceived quality (Group 2), the former achieved higher efficiency than the Group 2 airlines, which, on average, remained stable between 2011 and 2019 (Fig. 2a). In contrast, low perceived service quality carrier efficiency fell over the same period (year-by-year aggregated group-specific efficiencies and firm-level group-specific efficiencies are available in Tables A2 and A3, respectively, in the Appendix). In addition, Group 2 carriers’ efficiency presents a greater heterogeneity than that of Group 1 carriers (Fig. 2b). In this sense, we can conclude that during times not impacted by exogenous shocks, a tradeoff between technical efficiency and passenger satisfaction (perceived service quality) is not present for airlines.

The Group 1 firms that achieved the highest meta-technology efficiency compound annual growth rate (CAGR) were Qatar Airways (CAGR: 5.38%), China Southern Airlines (CAGR: 2.43%), and Air New Zealand (CAGR: 2.03%). The worst performers in the same group for the period 2011–2019 were Air France (CAGR: −5.29%), ANA ALL Nippon Airways (CAGR: −3.17%), and EasyJet (CAGR: −2.99%). Focusing on Group 2 airlines, Starflyer (CAGR: 3.16%), Air Europa (CAGR: 3.10%), and Regional Express (CAGR: 2.75%) reported the highest CAGR, while Juneyao Air (CAGR: −6.58%), Sichuan Airlines (CAGR: −6.30%), and Pegasus (CAGR: −4.02%) presented the most considerable deterioration in their efficiency over the period under review. In 2019, four out of five airlines with bias-corrected meta-efficiencies above 0.9 are regarded as high perceived quality services (Group 1) (i.e., United Airlines, Emirates, Qatar Airways, and Delta Airlines). In Group 2, only Alliance Airlines was assigned an efficiency of 0.9437, which was the highest in 2019 (Table A2 in the Appendix). This finding supports Swaminathan et al.‘s [81] finding that larger firms such as United Airlines, Emirates, Qatar Airways, and Delta Airlines have a higher likelihood of achieving high customer satisfaction and efficiency.

As discussed in the methodology section (expressions (21) and (22)), technology gap inefficiency expresses the distance between a firm's group-specific frontier and the meta-frontier. The greater the technology gap, the higher the inefficiency of a firm, which is attributed to exogenous factors such as macroeconomic conditions, national regulations, and cultural constraints affecting employment practices and decision-making. In addition, according to Scherage [82], the presence of technology gaps could be due to the ownership structure of airlines (e.g., state-owned vs. private), the intervention of governments in airlines' flight schedules (e.g., dictating services to destinations without financial benefit for the airlines), and operating cost subsidization to enhance the competitiveness of a firm against others while violating antitrust laws. Kounetas and Zervopoulos [105] link technology gap inefficiency to allocative inefficiency. In other words, inefficient pricing of goods and services toward consumers.

**Fig. 3:**
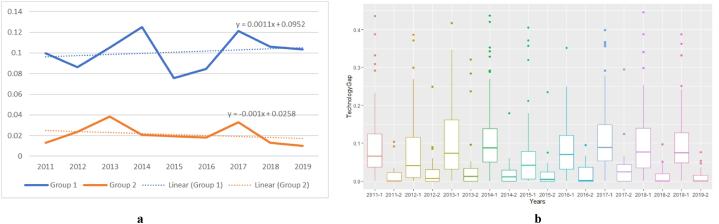
aTechnology gaps, Fig. 3b. Technology gaps boxplots Fig. 3. Technology gaps for high (Group 1) and low (Group 2) perceived quality services airlines.

Drawing on Fig. 3a and b (year-by-year firm-level technology gaps are available in Table A5 in the Appendix), Group 1 airlines reported higher technology gap inefficiencies than Group 2 airlines throughout the review period. Notably, only two of the 13 airlines reporting zero technology gap inefficiency in 2019 were from Group 1 (i.e., Emirates and United Airlines). The remaining were classified into Group 2 (i.e., Air Arabia, Air Canada, Edelweiss Air, Envoy Air, Jeju Air, Juneyao Air, Pakistan International Airlines, Skywest Airlines, Srilankan Airlines, Sun Country Airlines, and Ukraine International Airlines). According to Table A2, the highest efficiencies among the Group 2 airlines with a zero technology gap are assigned to Edelweiss Air (FSC/leisure) and Air Arabia (LCC).

Combining the findings in Fig. 2, Fig. 3, we conclude that customers seek high-quality services at reasonable prices when they are free from exogenous shocks. On average, airlines investing in service quality achieve higher technical efficiency and allocative inefficiency. An attractive pricing policy in conjunction with new cabin products (e.g., premium economy cabins) could be a key driver for reducing allocative inefficiency while retaining or improving technical efficiency and high perceived service quality.

### 5.1 Discussion

Our findings are in line with the stream of literature advocating the moderating role of airline strategy in the relationship between service quality and airline efficiency, and this relationship is extended to the pricing of airline services. Specifically, this study supports the conclusions drawn by Parast and Fini [37] and Mellat-Parast et al. [38] that a low perceived quality of services negatively impacts airline efficiency. According to the findings of this study, airlines classified as low-quality (not listed in the world's top 100) decreased efficiency over the review period. However, airlines offering high-quality services had a higher technical efficiency, which remained stable between 2011 and 2019.

One aspect neglected in the literature is airlines' allocative efficiency. Specifically, airline customers are price-sensitive. The technology gap inefficiency of high-quality airlines is considerably greater than that of their low-quality counterparts. Given the direct relationship between efficiency and firm profits [41], high-quality airlines can further increase their profits by finding a balance between service quality and cost. Premium economy cabins could be one of the features of this quality-price balance. Emphasizing carriers not listed in the world's top 100 service quality group and investing in quality of services such as legroom, inflight entertainment, and the Internet, which are deemed standardized, the tradeoff between quality and cost is less likely to apply.

## 6 Conclusions

In this study, we estimated the efficiencies and meta-technology efficiencies of 83 FSCs and LCCs between 2011 and 2019. Our sample comprises 71 FSCs and 12 LCCs. We classified these firms into two groups: (a) high-perceived quality airlines (47 airlines) and (b) low-perceived quality airlines (36 airlines). This classification drew on the Skytrax rating (i.e., high-perceived quality airlines were consistently ranked in the top 100 throughout the review period, while those that did not meet this criterion were regarded as low-perceived quality airlines). The operational data for estimating efficiency were sourced from the ICAO Data + database. Given the unbalanced design of the two groups and to prevent efficiency measurement bias due to the sample size and dimension of the input-output space, we applied a novel Bayesian generalized directional distance function approach to obtain group-specific and meta-technology efficiency estimates. The estimates obtained from this method were consistent with a considerably lower bias than those of other extant approaches. A detailed discussion of the Bayesian DEA approach and performance of its estimates is available in Ref. [33].

Our analysis aims to support decision-making toward efficiency and enhanced customer satisfaction in the airline industry. Our findings are based on data from a period free from considerable external shocks (e.g., the Global Financial Crisis, COVID-19). We found that LCCs were more efficient than FSCs, but their efficiencies converged during 2011–2019, mainly because of the efficiency deterioration of LCCs. Airlines deemed to have high service quality by customers also reported higher efficiency than those regarded as having low service quality. In this sense, there is no tradeoff between customer satisfaction and efficiency for most of the sample airlines. Acknowledging that this inverse relationship is either absent or weak in industries where standardization quality prevails (standardization quality mainly refers to products rather than services), airlines' passengers seem to value more tangibles (e.g., cabin configuration, seat comfort, ambiance, cleanliness, onboard meals, and technologies). Also, drawing on the literature about airline passengers’ satisfaction drivers, the firms classified as high service quality are perceived as “safer” than the remaining airlines. Although high-service-quality airlines are also more efficient, they report a higher technology gap inefficiency than their low-service-quality counterparts, possibly because of allocative inefficiencies. Based on our findings, during periods with or without minor impacts from external shocks, airlines could emphasize offering premium products at reasonable prices (e.g., premium economy cabins, meals, and inflight technologies), user-friendly websites, and advertising their safety standards.

A weakness of our study is the omission of the periods impacted by external shocks. Data unavailability was the main reason for exclusion. We intend to expand our analysis once operational and customer satisfaction data become available from 2020 onward for a sufficiently large sample of airlines. Although this extension would provide insightful managerial implications for challenging times, it would not limit the validity and generalizability of the findings of this study. To prevent distortions in airline efficiency estimations, separate analyses should be conducted for “normal” and challenging times of significant external shocks. Emphasizing the methodology, we could use a bias-corrected network data envelopment analysis (NDEA) model in conjunction with customers' perceptions, considering the internal structure of airlines’ operations, instead of a “black box” DEA model. Despite using a DEA approach that does not consider the internal structure of airlines, the obtained efficiencies are valid. The advantage of applying an NDEA model is the estimation of efficiency for each stage of airline activity (e.g., operations, services, and sales). To the best of our knowledge, no NDEA program in the literature provides explicit statistical inferences regarding efficiency. Extending the Bayesian approach used in this study to NDEA would be an appropriate direction for future research.

## Author contribution statement

Abdelrahman Rashid Bin Taliah; Panagiotis D Zervopoulos: Conceived and designed the experiments; Performed the experiments; Analyzed and interpreted the data; Contributed reagents, materials, analysis tools or data; Wrote the paper.

## Data availability statement

The authors do not have permission to share data.

## Declaration of competing interest

The authors declare that they have no known competing financial interests or personal relationships that could have appeared to influence the work reported in this paper.

## Figures and Tables

**Table A1:** Sample airlines

#	Group 1: High perceived quality services airlines			#	Group 2: Low perceived quality services airlines		
	Airlines	Type	Country		Airlines	Type	Country
1	Qatar Airways	FSC	Qatar	1	Kuwait Airways	FSC	Kuwait
2	Singapore Airlines (SIA)	FSC	Singapore	2	Iran Air	FSC	Iran
3	Asiana Airlines	FSC	South Korea	3	Regional Express (REX)	FSC	Australia
4	Etihad Airways	FSC	UAE	4	Star Flyer	FSC	Japan
5	Air New Zealand	FSC	New Zealand	5	Uzbekistan Airways	FSC	Uzbekistan
6	Qantas Airways	FSC	Australia	6	Middle East Airlines	FSC	Lebanon
7	Turkish Airlines	FSC	Turkey	7	Alliance Airlines	FSC	Australia
8	Emirates	FSC	UAE	8	Edelweiss Air	FSC (Leisure)	Switzerland
9	ANA ALL Nippon Airways	FSC	Japan	9	TUI fly Deutschland	FSC (Leisure)	Germany
10	Malaysia Airlines	FSC	Malaysia	10	Tigerair Taiwan	LCC	Republic of China (Taiwan)
11	Swiss International Airlines	FSC	Switzerland	11	Volotea	LCC	Spain
12	Lufthansa	FSC	Germany	12	Air Canada Express	FSC (Regional)	Canada
13	Eva Air	FSC	Republic of China (Taiwan)	13	Helvetic Airways	FSC (Regional)	Switzerland
14	Austrian Airlines	FSC	Austria	14	Air Europa	FSC	Spain
15	Garuda Indonesia	FSC	Indonesia	15	Sichuan Airlines	FSC	People's Republic of China
16	Air Canada	FSC	Canada	16	Jeju Air	LCC	South Korea
17	Aegean Airlines	FSC	Greece	17	Juneyao Air	FSC	People's Republic of China
18	Hainan Airlines	FSC	People's Republic of China	18	Shanghai Airlines	FSC	People's Republic of China
19	Korean Air	FSC	South Korea	19	Air Algerie	FSC	Algeria
20	China Southern Airlines	FSC	People's Republic of China	20	Tunisair	FSC	Tunisia
21	Air China	FSC	People's Republic of China	21	Envoy Air	FSC (Regional)	USA
22	Finnair	FSC	Finland	22	Ukraine International Airlines	FSC	Ukraine
23	South African Airways	FSC	South Africa	23	Skywest Airlines	FSC (Regional)	USA
24	British Airways	FSC	UK	24	Air Baltic	FSC	Latvia
25	KLM Royal Dutch Airlines	FSC	Netherlands	25	Pakistan International Airlines	FSC	Pakistan
26	Virgin Atlantic	FSC	UK	26	Volaris	LCC	Mexico
27	Vietnam Airlines	FSC	Vietnam	27	Sun Country Airlines	LCC	USA
28	Japan Airlines	FSC	Japan	28	Spirit Airlines	LCC	USA
29	Avianca	FSC	Colombia	29	Ryanair	LCC	Ireland
30	Hong Kong Airlines	FSC	Hong Kong	30	Norwegian Air Shuttle	LCC	Norway
31	Air France	FSC	France	31	SriLankan Airlines	FSC	Sri Lanka
32	Copa Airlines	FSC	Panama	32	Frontier Airlines	LCC	USA
33	Delta Airlines	FSC	USA	33	Transavia	LCC	Netherlands
34	Aeroflot	FSC	Russia	34	Pegasus Airlines	LCC	Turkey
35	Air Mauritius	FSC	Mauritius	35	Air Arabia	LCC	UAE
36	Alaska Airlines	FSC	USA	36	Jazeera Airways	LCC	Kuwait
37	Kenya Airways	FSC	Kenya				
38	United Airlines	FSC	USA				
39	Azul Airlines	LCC	Brazil				
40	American Airlines	FSC	USA				
41	SAS Scandinavian	FSC	Denmark, Norway, Sweden				
42	Air Astana	FSC	Kazakhstan				
43	Hawaiian Airlines	FSC	USA				
44	Gulf Air	FSC	Bahrain				
45	JetBlue Airways	LCC	USA				
46	EasyJet	LCC	Switzerland				
47	IndiGo	LCC	India				

**Table A2:** Firm-level meta-technology efficiencies

#	Airlines^1^	Type	2011	2012	2013	2014	2015	2016	2017	2018	2019	CAGR	St. Deviation
Group 1: High perceived quality services airlines													
1	Qatar Airways	FSC	0.5668	0.9063	0.9414	0.5653	0.9144	0.8954	0.7775	0.9325	0.9086	5.38%	0.1533
2	China Southern Airlines	FSC	0.5561	0.5248	0.6122	0.5661	0.7038	0.6019	0.6104	0.6302	0.6900	2.43%	0.0589
3	Air New Zealand	FSC	0.5689	0.5854	0.6275	0.5795	0.5989	0.5900	0.5596	0.6121	0.6814	2.03%	0.0368
4	Air Canada	FSC	0.6644	0.7615	0.7316	0.6736	0.8420	0.8957	0.7493	0.7163	0.7926	1.98%	0.0755
5	SAS Scandinavian	FSC	0.4989	0.5684	0.5679	0.5253	0.5396	0.5552	0.5902	0.5697	0.5828	1.74%	0.0293
6	Hainan Airlines	FSC	0.5361	0.5252	0.5981	0.5736	0.6271	0.6057	0.5717	0.6344	0.6225	1.67%	0.0394
7	Air Astana	FSC	0.4936	0.5394	0.5443	0.5127	0.5417	0.5147	0.5275	0.5390	0.5568	1.35%	0.0197
8	Copa Airlines	FSC	0.5601	0.5926	0.6684	0.5992	0.6440	0.9153	0.6255	0.6704	0.6289	1.29%	0.1036
9	Aeroflot	FSC	0.5734	0.5890	0.5990	0.5728	0.5999	0.6381	0.6136	0.5870	0.6393	1.22%	0.0247
10	Finnair	FSC	0.7132	0.6607	0.6860	0.7453	0.8648	0.7828	0.8194	0.8462	0.7854	1.08%	0.0711
11	Alaska Airlines	FSC	0.5200	0.9178	0.9093	0.5763	0.9074	0.9041	0.9455	0.5307	0.5708	1.04%	0.1948
12	United Airlines	FSC	0.8407	0.9033	0.9545	0.6495	0.9049	0.8739	0.6717	0.8849	0.9189	0.99%	0.1091
13	IndiGo	LCC	0.4555	0.4834	0.4866	0.4981	0.5185	0.4663	0.4680	0.4977	0.4931	0.89%	0.0195
14	Garuda Indonesia	FSC	0.6128	0.5975	0.6243	0.6481	0.6650	0.7051	0.6950	0.6863	0.6615	0.85%	0.0376
15	Malaysia Airlines	FSC	0.5503	0.5188	0.5446	0.5492	0.5979	0.5998	0.6104	0.6474	0.5839	0.66%	0.0403
16	Hong Kong Airlines	FSC	0.5931	0.5082	0.5600	0.5901	0.5883	0.6248	0.8827	0.7038	0.6287	0.65%	0.1083
17	Vietnam Airlines	FSC	0.5867	0.6254	0.5858	0.5933	0.6357	0.6494	0.6007	0.6108	0.6206	0.63%	0.0224
18	Asiana Airlines	FSC	0.6300	0.6122	0.6217	0.5803	0.6424	0.6668	0.6450	0.6893	0.6571	0.47%	0.0320
19	Gulf Air	FSC	0.7979	0.7071	0.7913	0.6913	0.7096	0.7415	0.7737	0.7919	0.8299	0.44%	0.0486
20	Virgin Atlantic	FSC	0.6589	0.6150	0.6052	0.6584	0.7618	0.6293	0.6018	0.6175	0.6746	0.26%	0.0503
21	Austrian Airlines	FSC	0.5660	0.6064	0.5471	0.5429	0.5619	0.6076	0.5663	0.5896	0.5770	0.21%	0.0234
22	Etihad Airways	FSC	0.6406	0.6310	0.9273	0.6042	0.6329	0.6309	0.6206	0.6124	0.6524	0.20%	0.1008
23	Air China	FSC	0.5252	0.5879	0.5304	0.5167	0.5563	0.5898	0.5309	0.5590	0.5337	0.18%	0.0270
24	Turkish Airlines	FSC	0.6439	0.6368	0.6212	0.6278	0.7741	0.6308	0.6316	0.6951	0.6519	0.14%	0.0490
25	Emirates	FSC	0.9070	0.8887	0.9198	0.9102	0.9135	0.8643	0.8940	0.9163	0.9176	0.13%	0.0182
26	Korean Air	FSC	0.6146	0.6385	0.6279	0.5975	0.6256	0.6331	0.6265	0.6809	0.6203	0.10%	0.0226
27	Swiss International Airlines	FSC	0.5147	0.4982	0.4749	0.4831	0.5063	0.5326	0.5237	0.5029	0.5153	0.01%	0.0185
28	JetBlue Airways	LCC	0.6492	0.7381	0.6774	0.6673	0.7300	0.6479	0.6389	0.6556	0.6486	−0.01%	0.0367
29	British Airways	FSC	0.6419	0.7282	0.6958	0.7184	0.8704	0.5520	0.6482	0.6517	0.6315	−0.18%	0.0882
30	American Airlines	FSC	0.8173	0.7197	0.6428	0.5101	0.7107	0.8616	0.5645	0.8289	0.7925	−0.34%	0.1229
31	Singapore Airlines (SIA)	FSC	0.8818	0.9322	0.8766	0.7445	0.9344	0.8119	0.7847	0.7952	0.8403	−0.53%	0.0664
32	Lufthansa	FSC	0.8307	0.8290	0.7935	0.9021	0.9376	0.7514	0.7261	0.8335	0.7774	−0.73%	0.0680
33	Delta Airlines	FSC	0.9742	0.9014	0.8772	0.8646	0.9364	0.9069	0.5844	0.8793	0.9072	−0.79%	0.1122
34	Air Mauritius	FSC	0.6751	0.6155	0.6079	0.6229	0.6210	0.7016	0.6223	0.6233	0.6275	−0.81%	0.0313
35	Avianca	FSC	0.5645	0.5910	0.5356	0.5082	0.5438	0.5014	0.4881	0.4962	0.5161	−0.99%	0.0344
36	Hawaiian Airlines	FSC	0.6727	0.6634	0.6778	0.6202	0.6787	0.6362	0.6671	0.6511	0.6147	−1.00%	0.0245
37	Aegean Airlines	FSC	0.6407	0.6771	0.6650	0.6431	0.7345	0.7467	0.6275	0.5963	0.5810	−1.08%	0.0562
38	Azul Airlines	LCC	0.5136	0.5306	0.5151	0.5052	0.5253	0.5140	0.5246	0.4819	0.4640	−1.12%	0.0219
39	South African Airways	FSC	0.6250	0.6083	0.6150	0.5738	0.6265	0.6005	0.5815	0.5376	0.5447	−1.52%	0.0331
40	KLM Royal Dutch Airlines	FSC	0.9073	0.9060	0.8532	0.6607	0.8883	0.7366	0.6828	0.7535	0.7860	−1.58%	0.0954
41	Qantas Airways	FSC	0.6768	0.5201	0.5141	0.5190	0.5249	0.5683	0.5416	0.5720	0.5685	−1.92%	0.0509
42	Japan Airlines	FSC	0.7110	0.6063	0.6238	0.5544	0.6024	0.6498	0.5799	0.6254	0.5776	−2.28%	0.0463
43	Eva Air	FSC	0.6616	0.6281	0.6638	0.5945	0.9346	0.5676	0.5496	0.5912	0.5363	−2.31%	0.1207
44	Kenya Airways	FSC	0.7009	0.6580	0.6435	0.6150	0.6660	0.6214	0.6141	0.5802	0.5571	−2.52%	0.0442
45	EasyJet	LCC	0.8576	0.8984	0.9182	0.9067	0.8956	0.7823	0.9153	0.7731	0.6529	−2.99%	0.0906
46	ANA ALL Nippon Airways	FSC	0.9474	0.9180	0.5807	0.5865	0.6084	0.6616	0.6364	0.5988	0.7091	−3.17%	0.1413
47	Air France	FSC	0.8785	0.7586	0.6738	0.5822	0.9063	0.6133	0.5763	0.5716	0.5388	−5.29%	0.1384
Group 2: Low perceived quality services airlines													
48	Starflyer	FSC	0.5886	0.5983	0.5823	0.5727	0.6114	0.6560	0.6901	0.6712	0.7791	3.16%	0.0672
49	Air Europa	FSC	0.5725	0.6481	0.6848	0.9424	0.8389	0.7483	0.7365	0.7676	0.7535	3.10%	0.1068
50	Regional Express (REX)	FSC (Regional)	0.6957	0.7860	0.7846	0.8301	0.7515	0.7417	0.6101	0.9185	0.8877	2.75%	0.0946
51	Ukraine international	FSC	0.5413	0.5379	0.6107	0.5828	0.6379	0.6515	0.6684	0.6652	0.6551	2.14%	0.0515
52	Jazeera	LCC	0.7416	0.6992	0.7402	0.7201	0.7246	0.8614	0.8288	0.8296	0.8936	2.09%	0.0712
53	Jeju Air	LCC	0.6086	0.6360	0.6711	0.7330	0.8778	0.6680	0.7396	0.6833	0.7174	1.84%	0.0783
54	Volaris	LCC	0.4953	0.5649	0.5288	0.5323	0.5327	0.5723	0.5426	0.5456	0.5523	1.22%	0.0226
55	Tunisair	FSC	0.4772	0.5102	0.5132	0.5137	0.5115	0.5143	0.5233	0.5296	0.5296	1.16%	0.0156
56	Middle East Airlines	FSC	0.5614	0.5807	0.5866	0.5661	0.5875	0.5877	0.5811	0.6038	0.6188	1.09%	0.0175
57	Kuwait Airways	FSC	0.4817	0.4869	0.5004	0.5152	0.5209	0.5315	0.5291	0.5178	0.5266	0.99%	0.0183
58	Envoy Air	FSC (Regional)	0.4428	0.4310	0.4934	0.4872	0.5037	0.8794	0.4415	0.4668	0.4740	0.76%	0.1395
59	Shanghai airlines	FSC	0.4449	0.4195	0.4627	0.4543	0.4696	0.4429	0.4369	0.4381	0.4712	0.64%	0.0170
60	Alliance Airlines	FSC	0.9098	0.9074	0.9299	0.9468	0.9192	0.9216	0.9354	0.9052	0.9437	0.41%	0.0155
61	Volotea	LCC	0.7351	0.6702	0.7351	0.8917	0.7662	0.7057	0.6984	0.7469	0.7601	0.37%	0.0630
62	Air Algerie	FSC	0.4770	0.4704	0.4582	0.4768	0.4702	0.5000	0.4596	0.4804	0.4917	0.34%	0.0137
63	Skywest Airlines	FSC (Regional)	0.4674	0.4906	0.5062	0.4831	0.4984	0.4838	0.4888	0.4840	0.4767	0.22%	0.0114
64	Uzbekistan Airways	FSC	0.5887	0.6017	0.6162	0.5764	0.6410	0.6190	0.6206	0.5930	0.6002	0.22%	0.0197
65	Air Arabia	LCC	0.8026	0.8619	0.8166	0.7384	0.8663	0.8568	0.8183	0.7875	0.8162	0.19%	0.0407
66	Pakistan International Airlines	FSC	0.5050	0.5177	0.4861	0.4809	0.4993	0.5354	0.4856	0.5114	0.4974	−0.17%	0.0175
67	Srilankan Airlines	FSC	0.5294	0.5513	0.5741	0.5282	0.5546	0.5469	0.5249	0.5359	0.5209	−0.18%	0.0174
68	Spirit Airlines	LCC	0.5633	0.5531	0.6010	0.5200	0.4968	0.5397	0.5351	0.5442	0.5496	−0.27%	0.0288
69	Helvetic Airways	FSC (Regional)	0.9234	0.8792	0.8880	0.8907	0.8570	0.9107	0.9622	0.8889	0.8995	−0.29%	0.0299
70	Ryanair	LCC	0.9214	0.8901	0.9026	0.9196	0.9221	0.8793	0.8924	0.9088	0.8925	−0.35%	0.0157
71	Edelweiss Air	FSC (Leisure)	0.9313	0.8988	0.9029	0.9162	0.8303	0.8604	0.9087	0.8573	0.8986	−0.40%	0.0327
72	Norwegian air Shuttle	LCC	0.9466	0.8656	0.8710	0.8762	0.8910	0.8925	0.7439	0.8724	0.8857	−0.74%	0.0536
73	Iran Air	FSC	0.5066	0.5252	0.5378	0.4911	0.4846	0.4979	0.4726	0.5046	0.4678	−0.88%	0.0230
74	Tigerair Taiwan	LCC	0.9590	0.9440	0.8184	0.7292	0.7687	0.7410	0.8355	0.8884	0.8771	−0.99%	0.0839
75	Air Canada Express	FSC (Regional)	0.8135	0.7025	0.6376	0.6351	0.6743	0.7681	0.6885	0.7101	0.7432	−1.00%	0.0590
76	Air Baltic	FSC	0.6415	0.5751	0.6207	0.5744	0.6326	0.6344	0.5678	0.5409	0.5456	−1.78%	0.0398
77	Transavia	LCC	0.8224	0.9239	0.9324	0.6806	0.9167	0.8927	0.7654	0.7238	0.6862	−1.99%	0.1046
78	Frontier Airlines	LCC	0.7048	0.7314	0.7517	0.7359	0.9071	0.8485	0.5672	0.5624	0.5714	−2.30%	0.1236
79	TUI fly Deutschland	FSC (Leisure)	0.7099	0.8880	0.9110	0.9340	0.9234	0.9426	0.6337	0.5532	0.5381	−3.03%	0.1717
80	Sun Country Airlines	LCC	0.9608	0.8898	0.8270	0.7233	0.6605	0.6533	0.6186	0.6087	0.6809	−3.75%	0.1266
81	Pegasus	LCC	0.8768	0.8605	0.6327	0.6349	0.6378	0.5607	0.5741	0.5979	0.6061	−4.02%	0.1188
82	Sichuan Airlines	FSC	0.9494	0.6495	0.5014	0.4870	0.5105	0.4909	0.5427	0.5148	0.5284	−6.30%	0.1487
83	Juneyao Air	FSC	0.9249	0.6199	0.5219	0.4860	0.4781	0.4825	0.5046	0.4828	0.5012	−6.58%	0.1452
	Average	FSC	0.6648	0.6613	0.6597	0.6268	0.6844	0.6704	0.6362	0.6506	0.6543	−0.18%	0.0173
	Average	LCC	0.7187	0.7427	0.7229	0.6878	0.7324	0.7150	0.6748	0.6769	0.6709	−0.76%	0.0273

1 The airlines are ranked from highest to the lowest compound annual growth rate (CAGR).

**Table A3:** Group-specific and meta-technology efficiencies

Year	Groups	Group-specific efficiency				Meta-technology efficiency				Technology gap			
		Mean	St. Deviation	Min	Max	Mean	St. Deviation	Min	Max	Mean	St. Deviation	Min	Max
2011	Group 1	0.7639	0.1408	0.5147	0.9792	0.6642	0.1348	0.4555	0.9742				
	Group 2	0.6976	0.1877	0.4449	0.9798	0.6845	0.1827	0.4428	0.9608				
	All					0.6732	0.1570	0.4428	0.9742	0.0615	0.0909	0.0000	0.4364
2012	Group 1	0.7596	0.1265	0.5383	0.9717	0.6736	0.1364	0.4834	0.9322				
	Group 2	0.6979	0.1761	0.4310	0.9511	0.6744	0.1630	0.4195	0.9440				
	All					0.6739	0.1477	0.4195	0.9440	0.0585	0.0889	0.0000	0.3873
2013	Group 1	0.7766	0.1237	0.5452	0.9868	0.6715	0.1348	0.4749	0.9545				
	Group 2	0.7052	0.1722	0.4582	0.9589	0.6670	0.1551	0.4582	0.9324				
	All					0.6695	0.1432	0.4582	0.9545	0.0757	0.0983	0.0000	0.4179
2014	Group 1	0.7451	0.1383	0.5146	0.9723	0.6198	0.1057	0.4831	0.9102				
	Group 2	0.6779	0.1737	0.4574	0.9645	0.6572	0.1664	0.4543	0.9468				
	All					0.6363	0.1362	0.4543	0.9468	0.0792	0.1014	0.0000	0.4373
2015	Group 1	0.7830	0.1356	0.5339	0.9814	0.7075	0.1436	0.5063	0.9376				
	Group 2	0.6912	0.1684	0.4781	0.9531	0.6718	0.1648	0.4696	0.9234				
	All					0.6918	0.1534	0.4696	0.9376	0.0507	0.0863	0.0000	0.4060
2016	Group 1	0.7608	0.1268	0.5494	0.9564	0.6761	0.1229	0.4663	0.9153				
	Group 2	0.6969	0.1645	0.4432	0.9500	0.6788	0.1595	0.4429	0.9426				
	All					0.6773	0.1393	0.4429	0.9426	0.0554	0.0677	0.0000	0.3519
2017	Group 1	0.7659	0.1401	0.5520	0.9757	0.6444	0.1119	0.4680	0.9455				
	Group 2	0.6722	0.1621	0.4433	0.9877	0.6393	0.1498	0.4369	0.9622				
	All					0.6422	0.1291	0.4369	0.9622	0.0825	0.0957	0.0000	0.3995
2018	Group 1	0.7669	0.1338	0.5387	0.9731	0.6607	0.1170	0.4819	0.9325				
	Group 2	0.6601	0.1668	0.4381	0.9854	0.6471	0.1532	0.4381	0.9185				
	All					0.6547	0.1335	0.4381	0.9325	0.0652	0.0901	0.0000	0.4459
2019	Group 1	0.7603	0.1354	0.5241	0.9846	0.6568	0.1188	0.4640	0.9189				
	Group 2	0.6671	0.1641	0.4712	0.9693	0.6571	0.1595	0.4678	0.9437				
	All					0.6569	0.1373	0.4640	0.9437	0.0623	0.0819	0.0000	0.3882
CAGR	Group 1	−0.05%				−0.13%							
CAGR	Group 2	−0.50%				−0.45%							
CAGR	All					−0.27%				0.15%			

Group 1: High perceived quality services airlines.

Group 2: Low perceived quality services airlines.

**Table A4:** Firm-level group-specific efficiencies

#	Airlines^1^	Type	2011	2012	2013	2014	2015	2016	2017	2018	2019	CAGR	St. Deviation
Group 1: High perceived quality services airlines													
1	Qatar Airways	FSC	0.5883	0.9063	0.9731	0.6856	0.9144	0.8954	0.7775	0.9647	0.9826	5.86%	0.1396
2	Air Canada	FSC	0.7368	0.7634	0.7316	0.9654	0.8420	0.8957	0.9752	0.8612	0.9201	2.50%	0.0938
3	Malaysia Airlines	FSC	0.5724	0.5561	0.6062	0.6111	0.6403	0.5998	0.6902	0.6474	0.7016	2.29%	0.0494
4	Hainan Airlines	FSC	0.5728	0.5383	0.6098	0.5736	0.6271	0.7116	0.6517	0.6772	0.6817	1.95%	0.0583
5	China Southern Airlines	FSC	0.6049	0.5546	0.6209	0.6156	0.7620	0.6019	0.9722	0.8828	0.7188	1.93%	0.1435
6	Copa Airlines	FSC	0.6257	0.6633	0.7457	0.7116	0.6869	0.9153	0.7661	0.7862	0.7236	1.63%	0.0839
7	Vietnam Airlines	FSC	0.6632	0.7187	0.6762	0.7294	0.7443	0.7428	0.6917	0.8625	0.7664	1.62%	0.0593
8	Finnair	FSC	0.7962	0.7891	0.8655	0.9332	0.8657	0.9319	0.9655	0.9078	0.9125	1.53%	0.0614
9	Aeroflot	FSC	0.6091	0.5992	0.6584	0.6428	0.6406	0.6817	0.6641	0.6211	0.6904	1.40%	0.0315
10	Turkish Airlines	FSC	0.6666	0.7053	0.7320	0.7363	0.8372	0.7788	0.7006	0.8143	0.7543	1.38%	0.0553
11	Air New Zealand	FSC	0.6450	0.6016	0.6553	0.6655	0.6299	0.6257	0.6236	0.6265	0.7192	1.22%	0.0341
12	Garuda Indonesia	FSC	0.6290	0.6760	0.7136	0.7014	0.7157	0.7351	0.7835	0.6993	0.6940	1.10%	0.0419
13	American Airlines	FSC	0.8334	0.7197	0.6428	0.5146	0.7107	0.9394	0.5738	0.8289	0.9035	0.90%	0.1467
14	Gulf Air	FSC	0.8854	0.8851	0.9389	0.9604	0.9210	0.9314	0.9412	0.9037	0.9567	0.86%	0.0283
15	Austrian Airlines	FSC	0.6175	0.6156	0.6520	0.6799	0.6855	0.6130	0.6601	0.6965	0.6625	0.78%	0.0318
16	Lufthansa	FSC	0.8360	0.8326	0.7935	0.9200	0.9652	0.8913	0.9517	0.9731	0.8962	0.77%	0.0638
17	Etihad Airways	FSC	0.6829	0.6861	0.9310	0.7181	0.6978	0.7000	0.6753	0.6891	0.7229	0.63%	0.0797
18	British Airways	FSC	0.6791	0.7551	0.7741	0.7938	0.8704	0.5836	0.7720	0.8115	0.7153	0.58%	0.0831
19	Asiana Airlines	FSC	0.6668	0.6445	0.6943	0.6539	0.6937	0.7487	0.7341	0.6948	0.6985	0.52%	0.0343
20	Air China	FSC	0.5789	0.6039	0.7686	0.5534	0.5890	0.6022	0.5656	0.5857	0.6019	0.43%	0.0635
21	EasyJet	LCC	0.9565	0.8984	0.9182	0.9067	0.9224	0.9049	0.9169	0.9182	0.9846	0.32%	0.0277
22	Aegean Airlines	FSC	0.9495	0.9461	0.9397	0.9723	0.9140	0.8538	0.9196	0.9369	0.9692	0.23%	0.0357
23	Swiss International Airlines	FSC	0.5147	0.5440	0.5452	0.5345	0.5356	0.5494	0.5520	0.5387	0.5241	0.20%	0.0121
24	SAS Scandinavian	FSC	0.6019	0.5927	0.6171	0.5541	0.5689	0.6332	0.6424	0.6418	0.6052	0.06%	0.0311
25	KLM Royal Dutch Airlines	FSC	0.9525	0.9060	0.8862	0.9022	0.9436	0.9321	0.9483	0.9309	0.9449	−0.09%	0.0236
26	Delta Airlines	FSC	0.9792	0.9279	0.8772	0.8646	0.9364	0.9564	0.9412	0.8793	0.9701	−0.10%	0.0424
27	United Airlines	FSC	0.9310	0.9033	0.9868	0.6901	0.9152	0.8739	0.7416	0.9531	0.9189	−0.15%	0.0987
28	Alaska Airlines	FSC	0.9564	0.9238	0.9093	0.9293	0.9074	0.9140	0.9697	0.9191	0.9341	−0.26%	0.0213
29	Air Mauritius	FSC	0.9004	0.8690	0.8632	0.9119	0.9413	0.9502	0.9006	0.9222	0.8686	−0.40%	0.0318
30	Korean Air	FSC	0.7071	0.7138	0.6809	0.6468	0.7155	0.7657	0.7094	0.7228	0.6751	−0.51%	0.0337
31	Virgin Atlantic	FSC	0.7087	0.6984	0.7805	0.7163	0.7861	0.7340	0.6703	0.6809	0.6762	−0.52%	0.0428
32	Emirates	FSC	0.9624	0.8887	0.9198	0.9537	0.9814	0.8643	0.9251	0.9358	0.9176	−0.53%	0.0363
33	Singapore Airlines (SIA)	FSC	0.9488	0.9717	0.9643	0.8786	0.9344	0.9187	0.9757	0.9619	0.8990	−0.60%	0.0342
34	Air Astana	FSC	0.6846	0.7477	0.7336	0.6879	0.7038	0.6333	0.6259	0.6298	0.6484	−0.60%	0.0456
35	Hong Kong Airlines	FSC	0.8844	0.8955	0.7584	0.9330	0.9455	0.7714	0.9183	0.8443	0.8308	−0.69%	0.0680
36	IndiGo	LCC	0.6061	0.7371	0.8155	0.9188	0.9244	0.6921	0.6161	0.5922	0.5554	−0.96%	0.1408
37	JetBlue Airways	LCC	0.7799	0.7534	0.7825	0.7140	0.7922	0.8362	0.7879	0.7289	0.7082	−1.07%	0.0420
38	Qantas Airways	FSC	0.6768	0.6283	0.6021	0.5915	0.5339	0.6159	0.5845	0.6731	0.6132	−1.09%	0.0441
39	ANA ALL Nippon Airways	FSC	0.9474	0.9180	0.9275	0.6279	0.6614	0.7087	0.6930	0.6751	0.8375	−1.36%	0.1289
40	Hawaiian Airlines	FSC	0.9054	0.7585	0.8245	0.7278	0.6986	0.7363	0.6825	0.7281	0.7845	−1.58%	0.0692
41	Kenya Airways	FSC	0.8522	0.8267	0.8847	0.7542	0.8440	0.7142	0.7257	0.7113	0.7112	−1.99%	0.0706
42	Eva Air	FSC	0.7238	0.7514	0.6954	0.7136	0.9346	0.6362	0.6432	0.6134	0.6039	−1.99%	0.1015
43	South African Airways	FSC	0.7425	0.6822	0.6711	0.6630	0.6265	0.6422	0.6602	0.6099	0.6175	−2.03%	0.0405
44	Azul Airlines	LCC	0.9012	0.9026	0.9331	0.9425	0.8974	0.8660	0.9242	0.9277	0.7151	−2.54%	0.0696
45	Japan Airlines	FSC	0.8633	0.8520	0.8737	0.6398	0.7189	0.6920	0.6641	0.6428	0.6633	−2.89%	0.0995
46	Air France	FSC	0.8785	0.7586	0.7279	0.6816	0.9063	0.6667	0.9441	0.6415	0.5813	−4.49%	0.1279
47	Avianca	FSC	0.8963	0.8908	0.6004	0.5952	0.5707	0.5703	0.5802	0.5513	0.5525	−5.23%	0.1417
			Group 2: Low perceived quality services airlines										
48	Starflyer	FSC	0.5886	0.5998	0.6117	0.6302	0.6114	0.6870	0.7119	0.7059	0.8254	3.83%	0.0767
49	Air Europa	FSC	0.5725	0.6662	0.9218	0.9424	0.8790	0.7483	0.7942	0.8027	0.7735	3.40%	0.1190
50	Jazeera	LCC	0.7504	0.7231	0.7731	0.7201	0.7342	0.8614	0.8288	0.8374	0.9693	2.88%	0.0825
51	Regional Express (REX)	FSC (Regional)	0.6957	0.8208	0.8364	0.8301	0.7515	0.7417	0.6637	0.9699	0.8877	2.75%	0.0965
52	Volaris	LCC	0.4953	0.5649	0.5313	0.5506	0.5561	0.5723	0.5879	0.5456	0.6051	2.25%	0.0320
53	Ukraine international	FSC	0.5657	0.5620	0.6290	0.5956	0.6379	0.6969	0.6684	0.6652	0.6551	1.64%	0.0472
54	Jeju Air	LCC	0.6381	0.7210	0.9555	0.9118	0.9531	0.6680	0.7808	0.6833	0.7174	1.31%	0.1263
55	Kuwait Airways	FSC	0.4817	0.5085	0.5004	0.5152	0.5209	0.5315	0.5560	0.5178	0.5266	0.99%	0.0207
56	Tunisair	FSC	0.5015	0.5135	0.5491	0.5137	0.5484	0.5508	0.5233	0.5296	0.5447	0.92%	0.0185
57	Middle East Airlines	FSC	0.5835	0.5956	0.5879	0.5661	0.5875	0.5877	0.6148	0.6038	0.6263	0.79%	0.0179
58	Air Algerie	FSC	0.4770	0.4704	0.4582	0.4768	0.5084	0.5031	0.5156	0.4996	0.5095	0.73%	0.0205
59	Shanghai airlines	FSC	0.4449	0.4464	0.4751	0.4574	0.4819	0.4432	0.4433	0.4381	0.4712	0.64%	0.0163
60	Envoy Air	FSC (Regional)	0.4556	0.4310	0.4934	0.4936	0.5198	0.8794	0.4456	0.4837	0.4740	0.44%	0.1376
61	Alliance Airlines	FSC	0.9098	0.9500	0.9419	0.9468	0.9192	0.9216	0.9877	0.9464	0.9437	0.41%	0.0228
62	Volotea	LCC	0.7351	0.6702	0.7351	0.9378	0.7662	0.7057	0.7365	0.7469	0.7601	0.37%	0.0745
63	Uzbekistan Airways	FSC	0.5887	0.6017	0.6162	0.5764	0.6410	0.6502	0.6470	0.5956	0.6002	0.22%	0.0271
64	Skywest Airlines	FSC (Regional)	0.4710	0.4906	0.5303	0.5018	0.4984	0.5206	0.4888	0.4853	0.4767	0.13%	0.0194
65	Spirit Airlines	LCC	0.5633	0.5940	0.6971	0.5753	0.5450	0.5397	0.5471	0.5442	0.5550	−0.17%	0.0496
66	Air Arabia	LCC	0.8317	0.9220	0.8236	0.7980	0.8663	0.8568	0.8416	0.8097	0.8162	−0.21%	0.0375
67	Iran Air	FSC	0.5066	0.5373	0.5378	0.4911	0.4846	0.4993	0.4976	0.5046	0.4946	−0.27%	0.0191
68	Helvetic Airways	FSC	0.9549	0.9180	0.9297	0.8907	0.8570	0.9490	0.9862	0.9854	0.9301	−0.29%	0.0420
69	Norwegian air Shuttle	LCC	0.9466	0.8955	0.8710	0.9095	0.8910	0.9404	0.8686	0.8724	0.9213	−0.30%	0.0296
70	Ryanair	LCC	0.9214	0.9304	0.9026	0.9196	0.9479	0.8793	0.8924	0.9485	0.8925	−0.35%	0.0249
71	Edelweiss Air	FSC (Leisure)	0.9374	0.8988	0.9146	0.9202	0.8303	0.9053	0.9087	0.8573	0.8986	−0.47%	0.0330
72	Srilankan Airlines	FSC	0.5629	0.5513	0.5741	0.5377	0.5546	0.5469	0.5642	0.5359	0.5209	−0.86%	0.0165
73	Pakistan International Airlines	FSC	0.5399	0.5177	0.5043	0.4914	0.5227	0.5429	0.4921	0.5283	0.4974	−0.91%	0.0198
74	Tigerair Taiwan	LCC	0.9590	0.9440	0.8437	0.7580	0.7776	0.7919	0.8813	0.9243	0.8771	−0.99%	0.0740
75	Air Baltic	FSC	0.6415	0.5751	0.6515	0.5914	0.6369	0.6816	0.5953	0.5409	0.5591	−1.52%	0.0470
76	Transavia	LCC	0.8224	0.9239	0.9324	0.7073	0.9167	0.8927	0.7654	0.7715	0.6862	−1.99%	0.0961
77	Air Canada Express	FSC (Regional)	0.9049	0.9511	0.9589	0.6591	0.9087	0.8634	0.7539	0.7101	0.7432	−2.16%	0.1124
78	Frontier Airlines	LCC	0.7048	0.7314	0.7606	0.7909	0.9071	0.9123	0.5672	0.5624	0.5714	−2.30%	0.1366
79	TUI fly Deutschland	FSC (Leisure)	0.7099	0.8880	0.9110	0.9645	0.9371	0.9500	0.9285	0.5532	0.5536	−2.72%	0.1697
80	Sun Country Airlines	LCC	0.9658	0.8898	0.8680	0.7539	0.6900	0.6691	0.6395	0.6093	0.6809	−3.81%	0.1260
81	Pegasus	LCC	0.9798	0.9499	0.6426	0.6498	0.6822	0.5607	0.5741	0.5979	0.6126	−5.08%	0.1581
82	Sichuan Airlines	FSC	0.9530	0.6495	0.5129	0.4870	0.5314	0.4909	0.5427	0.5257	0.5308	−6.29%	0.1476
83	Juneyao Air	FSC	0.9461	0.6267	0.5686	0.4860	0.4781	0.4825	0.5480	0.4828	0.5012	−6.82%	0.1503

1 The airlines are ranked from highest to the lowest compound annual growth rate (CAGR).

**Table A5:** Airlines technology gaps

#	Airlines^1^	Type	Group	2011	2012	2013	2014	2015	2016	2017	2018	2019	CAGR	St. Deviation
1	Air Arabia	LCC	2	0.0291	0.0601	0.0070	0.0596	0.0000	0.0000	0.0233	0.0222	0.0000	−100.00%	0.0239
2	Air Canada Express	FSC (Regional)	2	0.0914	0.2487	0.3213	0.0240	0.2344	0.0953	0.0654	0.0000	0.0000	−100.00%	0.1186
3	Edelweiss Air	FSC (Leisure)	2	0.0061	0.0000	0.0117	0.0040	0.0000	0.0449	0.0000	0.0000	0.0000	−100.00%	0.0146
4	Emirates	FSC	1	0.0554	0.0000	0.0000	0.0435	0.0679	0.0000	0.0310	0.0195	0.0000	−100.00%	0.0266
5	Envoy Air	FSC (Regional)	2	0.0129	0.0000	0.0000	0.0064	0.0161	0.0000	0.0041	0.0169	0.0000	−100.00%	0.0072
6	Jeju Air	LCC	2	0.0295	0.0850	0.2844	0.1788	0.0754	0.0000	0.0412	0.0000	0.0000	−100.00%	0.0967
7	Juneyao Air	FSC	2	0.0212	0.0068	0.0467	0.0000	0.0000	0.0000	0.0434	0.0000	0.0000	−100.00%	0.0194
8	Pakistan International Airlines	FSC	2	0.0349	0.0000	0.0181	0.0105	0.0235	0.0075	0.0066	0.0169	0.0000	−100.00%	0.0114
9	Skywest Airlines	FSC (Regional)	2	0.0036	0.0000	0.0241	0.0187	0.0000	0.0368	0.0000	0.0013	0.0000	−100.00%	0.0137
10	Srilankan Airlines	FSC	2	0.0335	0.0000	0.0000	0.0095	0.0000	0.0000	0.0393	0.0000	0.0000	−100.00%	0.0158
11	Sun Country Airlines	LCC	2	0.0050	0.0000	0.0410	0.0306	0.0295	0.0158	0.0209	0.0006	0.0000	−100.00%	0.0155
12	Ukraine International Airlines	FSC	2	0.0244	0.0241	0.0183	0.0128	0.0000	0.0454	0.0000	0.0000	0.0000	−100.00%	0.0158
13	United Airlines	FSC	1	0.0903	0.0000	0.0323	0.0406	0.0103	0.0000	0.0699	0.0681	0.0000	−100.00%	0.0348
14	Virgin Atlantic	FSC	1	0.0498	0.0834	0.1753	0.0579	0.0243	0.1047	0.0685	0.0633	0.0016	−31.95%	0.0498
15	Pegasus	LCC	2	0.1030	0.0894	0.0099	0.0149	0.0444	0.0000	0.0000	0.0000	0.0066	−26.35%	0.0402
16	Avianca	FSC	1	0.3318	0.2998	0.0648	0.0870	0.0269	0.0689	0.0920	0.0551	0.0363	−21.79%	0.1143
17	SAS Scandinavian	FSC	1	0.1030	0.0243	0.0491	0.0288	0.0293	0.0779	0.0521	0.0722	0.0225	−15.56%	0.0282
18	Middle East Airlines	FSC	2	0.0222	0.0150	0.0013	0.0000	0.0000	0.0000	0.0337	0.0000	0.0076	−11.28%	0.0123
19	IndiGo	LCC	1	0.1506	0.2537	0.3289	0.4208	0.4060	0.2258	0.1481	0.0944	0.0623	−9.34%	0.1310
20	JetBlue Airways	LCC	1	0.1307	0.0153	0.1051	0.0467	0.0622	0.1884	0.1490	0.0733	0.0596	−8.35%	0.0553
21	Air Astana	FSC	1	0.1911	0.2084	0.1893	0.1752	0.1622	0.1186	0.0984	0.0908	0.0916	−7.85%	0.0473
22	Air New Zealand	FSC	1	0.0761	0.0162	0.0278	0.0860	0.0311	0.0357	0.0640	0.0145	0.0378	−7.48%	0.0259
23	Japan Airlines	FSC	1	0.1523	0.2457	0.2499	0.0854	0.1165	0.0422	0.0842	0.0175	0.0857	−6.19%	0.0821
24	China Southern Airlines	FSC	1	0.0488	0.0299	0.0087	0.0495	0.0581	0.0000	0.3618	0.2526	0.0287	−5.72%	0.1258
25	Korean Air	FSC	1	0.0924	0.0753	0.0530	0.0492	0.0899	0.1326	0.0829	0.0419	0.0548	−5.64%	0.0286
26	South African Airways	FSC	1	0.1176	0.0739	0.0562	0.0892	0.0000	0.0418	0.0786	0.0723	0.0728	−5.19%	0.0327
27	Tunisair	FSC	2	0.0243	0.0033	0.0359	0.0000	0.0370	0.0365	0.0000	0.0000	0.0152	−5.09%	0.0168
28	Azul Airlines	LCC	1	0.3877	0.3720	0.4179	0.4373	0.3720	0.3519	0.3995	0.4459	0.2510	−4.71%	0.0581
29	Sichuan Airlines	FSC	2	0.0036	0.0000	0.0115	0.0000	0.0210	0.0000	0.0000	0.0109	0.0024	−4.47%	0.0074
30	Hong Kong Airlines	FSC	1	0.2913	0.3873	0.1984	0.3429	0.3572	0.1465	0.0356	0.1405	0.2022	−3.98%	0.1182
31	Hawaiian Airlines	FSC	1	0.2328	0.0952	0.1467	0.1075	0.0199	0.1001	0.0154	0.0770	0.1698	−3.45%	0.0693
32	Alaska Airlines	FSC	1	0.4364	0.0060	0.0000	0.3530	0.0000	0.0099	0.0243	0.3883	0.3633	−2.02%	0.2002
33	Singapore Airlines (SIA)	FSC	1	0.0670	0.0395	0.0877	0.1341	0.0000	0.1068	0.1910	0.1666	0.0586	−1.47%	0.0615
34	Helvetic Airways	FSC (Regional)	2	0.0315	0.0388	0.0417	0.0000	0.0000	0.0383	0.0241	0.0965	0.0307	−0.30%	0.0284
35	Kenya Airways	FSC	1	0.1513	0.1688	0.2412	0.1392	0.1779	0.0928	0.1116	0.1311	0.1540	0.20%	0.0428
36	Air Mauritius	FSC	1	0.2253	0.2535	0.2553	0.2890	0.3203	0.2486	0.2783	0.2988	0.2412	0.76%	0.0307
37	Eva Air	FSC	1	0.0622	0.1233	0.0316	0.1191	0.0000	0.0686	0.0936	0.0222	0.0677	0.95%	0.0423
38	Asiana Airlines	FSC	1	0.0369	0.0323	0.0725	0.0736	0.0514	0.0820	0.0891	0.0055	0.0415	1.31%	0.0274
39	Aegean Airlines	FSC	1	0.3087	0.2691	0.2747	0.3292	0.1795	0.1071	0.2921	0.3406	0.3882	2.58%	0.0857
40	Air China	FSC	1	0.0537	0.0160	0.2382	0.0367	0.0326	0.0124	0.0347	0.0267	0.0681	2.68%	0.0699
41	Aeroflot	FSC	1	0.0358	0.0102	0.0594	0.0700	0.0407	0.0436	0.0504	0.0341	0.0511	4.04%	0.0170
42	Copa Airlines	FSC	1	0.0656	0.0706	0.0772	0.1124	0.0429	0.0000	0.1406	0.1158	0.0948	4.18%	0.0423
43	Gulf Air	FSC	1	0.0875	0.1780	0.1477	0.2691	0.2115	0.1899	0.1675	0.1118	0.1268	4.20%	0.0552
44	Finnair	FSC	1	0.0830	0.1284	0.1795	0.1879	0.0009	0.1491	0.1461	0.0616	0.1271	4.85%	0.0599
45	Hainan Airlines	FSC	1	0.0367	0.0131	0.0117	0.0000	0.0000	0.1058	0.0800	0.0428	0.0592	5.44%	0.0371
46	Austrian Airlines	FSC	1	0.0515	0.0092	0.1049	0.1370	0.1235	0.0054	0.0938	0.1069	0.0855	5.80%	0.0476
47	Etihad Airways	FSC	1	0.0423	0.0551	0.0037	0.1139	0.0649	0.0691	0.0547	0.0766	0.0705	5.85%	0.0294
48	Air Canada	FSC	1	0.0724	0.0019	0.0000	0.2918	0.0000	0.0000	0.2258	0.1449	0.1275	6.50%	0.1093
49	Vietnam Airlines	FSC	1	0.0765	0.0932	0.0904	0.1361	0.1086	0.0934	0.0910	0.2517	0.1458	7.44%	0.0541
50	Garuda Indonesia	FSC	1	0.0162	0.0786	0.0893	0.0533	0.0507	0.0300	0.0885	0.0130	0.0325	8.05%	0.0297
51	British Airways	FSC	1	0.0372	0.0269	0.0784	0.0754	0.0000	0.0316	0.1237	0.1598	0.0837	9.44%	0.0507
52	EasyJet	LCC	1	0.0989	0.0000	0.0000	0.0000	0.0268	0.1227	0.0015	0.1451	0.3318	14.39%	0.1107
53	Qatar Airways	FSC	1	0.0215	0.0000	0.0317	0.1204	0.0000	0.0000	0.0000	0.0322	0.0741	14.74%	0.0415
54	KLM Royal Dutch Airlines	FSC	1	0.0452	0.0000	0.0331	0.2415	0.0553	0.1956	0.2655	0.1774	0.1589	14.99%	0.0983
55	Turkish Airlines	FSC	1	0.0227	0.0685	0.1108	0.1085	0.0631	0.1480	0.0690	0.1192	0.1023	18.18%	0.0376
56	Malaysia Airlines	FSC	1	0.0220	0.0373	0.0616	0.0620	0.0424	0.0000	0.0797	0.0000	0.1178	20.48%	0.0382
57	American Airlines	FSC	1	0.0160	0.0000	0.0000	0.0045	0.0000	0.0778	0.0093	0.0000	0.1110	24.00%	0.0410
58	Jazeera	LCC	2	0.0088	0.0238	0.0330	0.0000	0.0096	0.0000	0.0000	0.0078	0.0757	27.01%	0.0245
59	Delta Airlines	FSC	1	0.0050	0.0265	0.0000	0.0000	0.0000	0.0495	0.3568	0.0000	0.0629	32.43%	0.1154
60	Lufthansa	FSC	1	0.0054	0.0036	0.0000	0.0179	0.0277	0.1399	0.2256	0.1396	0.1188	41.07%	0.0822
61	Air Algerie	FSC	2	0.0000	0.0000	0.0000	0.0000	0.0382	0.0030	0.0560	0.0192	0.0178	N/A	0.0203
62	Air Baltic	FSC	2	0.0000	0.0000	0.0308	0.0170	0.0043	0.0472	0.0275	0.0000	0.0135	N/A	0.0167
63	Air Europa	FSC	2	0.0000	0.0181	0.2370	0.0000	0.0401	0.0000	0.0577	0.0352	0.0200	N/A	0.0746
64	Air France	FSC	1	0.0000	0.0000	0.0541	0.0993	0.0000	0.0534	0.3678	0.0699	0.0425	N/A	0.1147
65	Alliance Airlines	FSC	2	0.0000	0.0426	0.0120	0.0000	0.0000	0.0000	0.0523	0.0412	0.0000	N/A	0.0222
66	ANA ALL Nippon Airways	FSC	1	0.0000	0.0000	0.3468	0.0414	0.0530	0.0471	0.0566	0.0763	0.1284	N/A	0.1061
67	Frontier Airlines	LCC	2	0.0000	0.0000	0.0089	0.0550	0.0000	0.0638	0.0000	0.0000	0.0000	N/A	0.0259
68	Iran Air	FSC	2	0.0000	0.0122	0.0000	0.0000	0.0000	0.0014	0.0250	0.0000	0.0267	N/A	0.0113
69	Kuwait Airways	FSC	2	0.0000	0.0215	0.0000	0.0000	0.0000	0.0000	0.0269	0.0000	0.0000	N/A	0.0108
70	Norwegian air Shuttle	LCC	2	0.0000	0.0298	0.0000	0.0333	0.0000	0.0478	0.1247	0.0000	0.0355	N/A	0.0402
71	Qantas Airways	FSC	1	0.0000	0.1083	0.0880	0.0725	0.0090	0.0476	0.0428	0.1012	0.0447	N/A	0.0384
72	Regional Express (REX)	FSC (Regional)	2	0.0000	0.0348	0.0519	0.0000	0.0000	0.0000	0.0536	0.0514	0.0000	N/A	0.0258
73	Ryanair	LCC	2	0.0000	0.0403	0.0000	0.0000	0.0258	0.0000	0.0000	0.0397	0.0000	N/A	0.0181
74	Shanghai airlines	FSC	2	0.0000	0.0270	0.0124	0.0031	0.0124	0.0003	0.0065	0.0000	0.0000	N/A	0.0091
75	Spirit Airlines	LCC	2	0.0000	0.0410	0.0960	0.0553	0.0482	0.0000	0.0120	0.0000	0.0053	N/A	0.0337
76	Star Flyer	FSC	2	0.0000	0.0014	0.0294	0.0576	0.0000	0.0309	0.0218	0.0346	0.0464	N/A	0.0209
77	Swiss International Airlines	FSC	1	0.0000	0.0458	0.0703	0.0514	0.0293	0.0169	0.0283	0.0358	0.0088	N/A	0.0220
78	Tigerair Taiwan	LCC	2	0.0000	0.0000	0.0253	0.0289	0.0088	0.0509	0.0458	0.0359	0.0000	N/A	0.0202
79	Transavia	LCC	2	0.0000	0.0000	0.0000	0.0267	0.0000	0.0000	0.0000	0.0476	0.0000	N/A	0.0172
80	TUI fly Deutschland	FSC (Leisure)	2	0.0000	0.0000	0.0000	0.0304	0.0136	0.0074	0.2947	0.0000	0.0155	N/A	0.0960
81	Uzbekistan Airways	FSC	2	0.0000	0.0000	0.0000	0.0000	0.0000	0.0312	0.0265	0.0026	0.0000	N/A	0.0126
82	Volaris	LCC	2	0.0000	0.0000	0.0025	0.0183	0.0234	0.0000	0.0452	0.0000	0.0528	N/A	0.0208
83	Volotea	LCC	2	0.0000	0.0000	0.0000	0.0462	0.0000	0.0000	0.0382	0.0000	0.0000	N/A	0.0187

Group 1: High perceived quality services airlines.

Group 2: Low perceived quality services airlines.

1 The airlines are ranked from lowest to the highest compound annual growth rate (CAGR).

## Footnotes

1 Expressions: (5), (6), (7), (8), (9), (10), (11), (12), (13), (14), (15), (16), (17), (18), (19), and (20).
